# MRI evaluation of cerebral perivascular spaces predicts amyloid-related imaging abnormalities risk in preclinical Alzheimer's disease

**DOI:** 10.3389/frdem.2026.1719740

**Published:** 2026-04-09

**Authors:** Bavrina Bigjahan, Michele Cavallari, Giuseppe Barisano

**Affiliations:** 1 School of Medicine, University of Illinois-Chicago, Chicago, IL, United States; 2 Department of Radiology, Keck School of Medicine, University of Southern California, Los Angeles, CA, United States; 3 Aging Brain Center, Marcus Institute for Aging Research, Hebrew SeniorLife, Harvard Medical School, Boston, MA, United States; 4 Division of Gerontology, Beth Israel Deaconess Medical Center, Harvard Medical School, Boston, MA, United States; 5 Quantitative Sciences Unit, Department of Medicine, Stanford University, Stanford, CA, United States

**Keywords:** Alzheimer's disease, amyloid-related imaging abnormalities, cerebral microbleeds, MRI, perivascular spaces, white matter hyperintensities, superficial siderosis, ARIA

## Abstract

**Background and purpose:**

Amyloid-related imaging abnormalities (ARIA) are radiographic findings observed in the natural course of Alzheimer's disease and have been reported at higher rates in patients receiving anti-amyloid monoclonal antibody therapy. Identifying novel radiographic factors predicting ARIA risk may help prevent its occurrence, improve patient stratification, and provide insight on the underlying biological mechanisms. It remains unclear whether cerebral perivascular spaces (PVS) along with other quantitative radiographic markers of cerebral small vessel disease may help predict the risk of incident ARIA in patients diagnosed with preclinical Alzheimer's disease.

**Methods:**

Participants from the A4 study were included. PVS and white matter hyperintensities (WMH) were segmented with robust fully-automated methods on T1-weighted and FLAIR images, respectively. Number of microhemorrhages and subcortical infarcts were previously recorded by expert radiologists. Baseline measurements of these markers were used in Cox proportional-hazards models to predict ARIA risk controlling for relevant demographic, clinical, and radiographic factors.

**Results:**

Among 6,028 brain MRI from 1,088 participants (median age: 71-y.o.; 59.4% women), 356 ARIA were diagnosed (median study follow-up: 5.4 years). The volume fraction of PVS and WMH, and the number of microhemorrhages at baseline predicted higher ARIA risk (adjusted hazard ratio ranges: 1.32–1.55; adjusted *p*-values all <0.05). Importantly, the effect of PVS on ARIA with microhemorrhages risk was observed in individuals considered at low risk of ARIA according to current guidelines, i.e., APOE-ε4 non-carriers, low WMH burden, or no microhemorrhages.

**Conclusions:**

These results support the use of quantitative measurements of PVS in addition to WMH and microhemorrhages to assist clinicians in estimating an individual's risk of ARIA.

## Introduction

Amyloid-related imaging abnormalities (ARIA) are a spectrum of findings on brain MRI related to the increased permeability of amyloid-laden blood vessels to fluid or blood products that can occur spontaneously in the setting of cerebral amyloid angiopathy (CAA) and as a result of the mobilization of amyloid-lowering monoclonal antibodies (Sperling et al., 2011). ARIA include two classes of MRI signal alterations: ARIA-E, which refers to parenchymal edema and sulcal effusion manifesting as hyperintensities on T2-weighted MRI sequences, without restricted signal on diffusion-weighted imaging (Sperling et al., 2011); ARIA-H, which refers to parenchymal microhemorrhages and superficial siderosis in the leptomeninges manifesting as hypointense signal on susceptibility-weighted imaging (Sperling et al., 2011). The pathophysiological mechanisms underlying ARIA are not fully understood, but it is hypothesized that perivascular inflammation related to the presence and/or removal of amyloid plaques may result in increased vascular fragility and leakage of proteinaceous fluid and blood products (Sperling et al., 2011). Although ARIA is often asymptomatic, severe neurological symptoms may occur in some cases, and death has been reported in rare instances (Filippi et al., 2022; Sims et al., 2023; van Dyck et al., 2023). Given the rise in the use of anti-amyloid molecules in clinical trials and their ongoing translation into clinical practice, identifying clinical factors and imaging markers that can predict an individual's risk of developing ARIA is critical for patient safety. Previous studies reported higher risk of ARIA in individuals carrying the ε4 allele of the Apolipoprotein-E gene (APOE4; Arrighi et al., 2016; Salloway et al., 2022; Sperling et al., 2012), and Apolipoprotein-E genotyping before treatment is currently recommended (Cummings et al., 2023). Obtaining a brain MRI scan within 3–4 months of beginning treatment to assess cerebrovascular health is also recommended (Cummings et al., 2023), as cerebrovascular disease may predispose to ARIA.

Perivascular spaces (PVS), also known as Virchow–Robin spaces, and white matter hyperintensities (WMH) are common MRI markers of cerebral small vessel disease and brain aging (Duering et al., 2023). PVS are fluid-filled spaces that surround penetrating arterioles and venules as they traverse the brain parenchyma and are visible on T1-weighted MRI as small, linear or punctate hypointense structures following the course of penetrating vessels. They are thought to play a key role in interstitial fluid drainage and glymphatic clearance (Wardlaw et al., 2020). WMH, typically detected on T2-weighted and FLAIR sequences, appear as areas of increased signal within the white matter and are generally interpreted as reflecting chronic small vessel–related injury, including demyelination, gliosis, and axonal loss (Wardlaw et al., 2015).

Currently it remains unclear whether quantitative measurements of MRI-visible perivascular spaces (PVS) are associated with higher risk of ARIA.

Here, the relation between the risk of ARIA and multiple quantitative markers of cerebral small vessel disease (SVD), including PVS, white matter hyperintensities (WMH), microhemorrhages, and subcortical infarcts, was investigated, while controlling for Apolipoprotein-E genotype and potential confounding cardiovascular risk factors in participants with preclinical Alzheimer's Disease enrolled in a phase-3 trial of solanezumab. Given that solanezumab showed relatively low efficacy in reducing amyloid-β burden (Sperling et al., 2023), our results refer to spontaneous ARIA and may not be directly generalizable to ARIA associated with the clearance of amyloid. Their role across subgroups was also assessed to identify effect modifications related to clinical, demographic, and radiological characteristics.

## Methods

### Study population

The Anti-Amyloid Treatment in Asymptomatic Alzheimer's Disease (A4) study was a phase-3, double-blind, placebo-controlled trial of solanezumab in patients with preclinical Alzheimer's disease conducted between 2014 and 2022 (Sperling et al., 2023). Candidates for the trial were self-referred individuals from the community. Recruitment strategies included community outreach, national and local advertising, and interrogation of national and local registries. Eligibility criteria in the first screening visit, which included 6,763 individuals, were: age between 65 and 85, living independently without a diagnosis of mild cognitive impairment or dementia, having a partner who provided information about the participant's functioning, and absence of cognitive impairment confirmed with a global Clinical Dementia Rating score of 0, a Mini–Mental State Examination score >24, and a Wechsler Memory Scale Logical Memory Delayed Recall score of 6–18. Persons with unstable medical conditions or use of medications for Alzheimer's Disease were excluded.

A total of 1,165 participants were considered eligible after ^18^F-florbetapir PET imaging (SUVR ≥1.15, or SUVR of ≥1.10 to <1.15 accompanied by a positive visual reading from a two-reader consensus determination) and were randomly assigned in a 1:1 ratio to receive intravenous solanezumab at a dose of up to 1600 mg intravenously every 4 weeks or placebo. Randomization was stratified according to APOE4 status, years of education ( ≤ 12 or >12), and trial site (Sperling et al., 2023).

Tau status was determined in a subset of 509 participants who underwent ^18^F-flortaucipir PET imaging (*N* = 365) and/or a lumbar puncture (*N* = 256) for the assessment of total tau and phosphorylated tau_181_ with enzyme-linked immunosorbent assay. These participants were classified as tau-positive based on abnormal level of total tau (>412 pg/ml; Fagan et al., 2011) or phosphorylated tau_181_ (>78 pg/ml; Fagan et al., 2011) or tau SUVR (>1.34 in the temporal meta-region of interest; Ossenkoppele et al., 2018).

Written informed consent was obtained from all participants. The Institutional Review Board at Stanford University (IRB-79206) approved this study, whereas approval from an institutional review board was obtained at each of the trial sites for conducting the trial (Sperling et al., 2023).

### MRI data acquisition and processing

**Figure 1:**
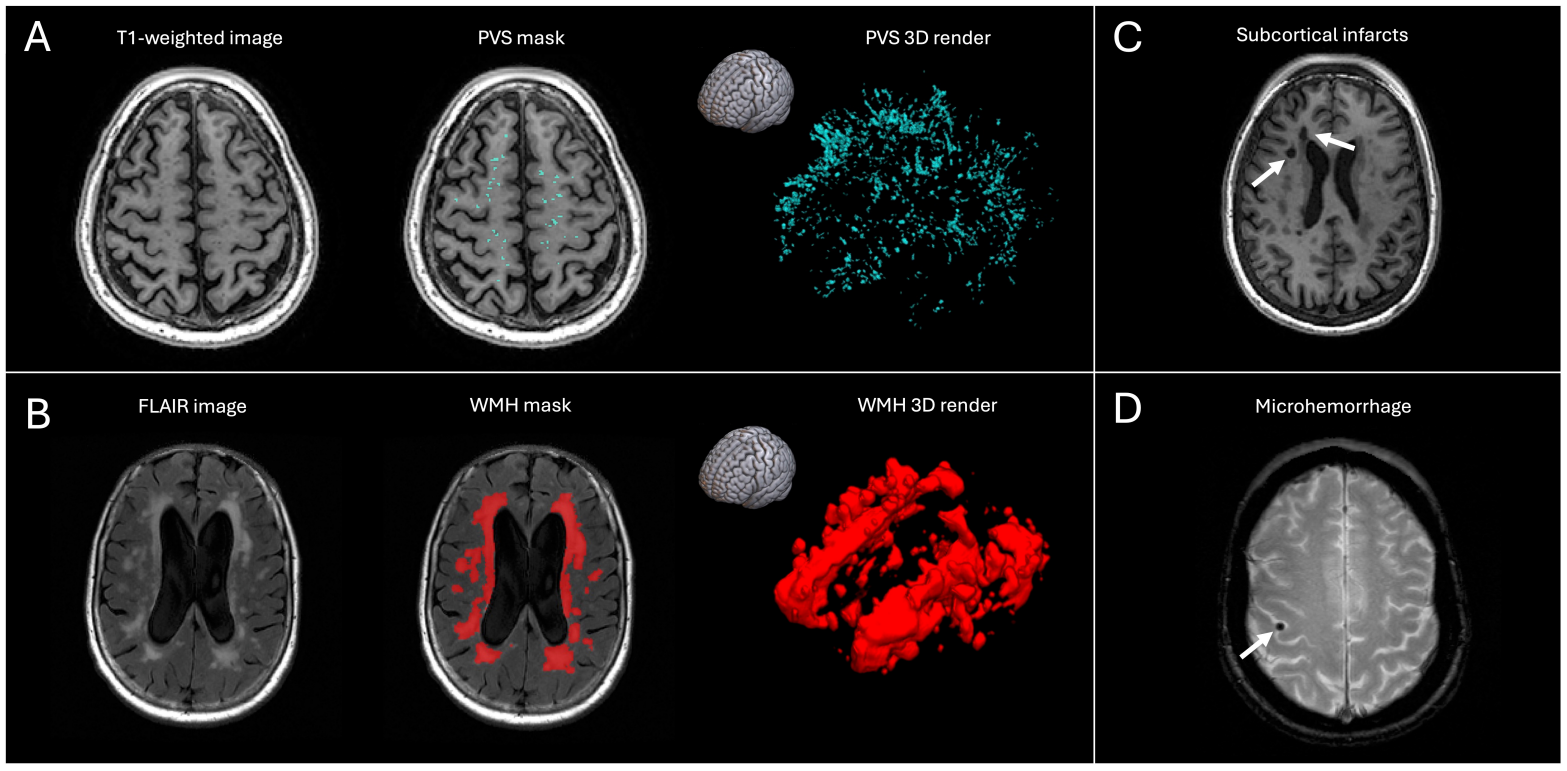
Representative images of cerebral small vessel disease radiographic markers assessed in this study. **(A)** Axial T1-weighted image without and with overlaid PVS mask in cyan. The image on the right is the corresponding 3D rendering of the PVS masks; the brain icon indicates the orientation of the 3D rendering. **(B)** Axial FLAIR image without and with overlaid WMH mask in red. The image on the right is the corresponding 3D rendering of the WMH mask; the brain icon indicates the orientation of the 3D rendering. **(C)** Axial T1-weighted image at baseline of a participant with subcortical infarcts (white arrows). **(D)** Axial T2-star image at baseline of a participant with a microhemorrhage (white arrow).

Brain MRI data were acquired with 3-Tesla scanners. Details about the scanners, sequences, and parameters of the images analyzed in this study are reported in Supplementary Table 1. The following MRI markers of cerebral small vessel disease were considered: microhemorrhages, subcortical infarcts, white matter hyperintensities (WMH), and perivascular spaces (PVS; Figure 1). A central read of the MRI data was performed for all subjects in a blinded fashion by experienced radiologists at the Mayo Clinic Aging and Dementia Imaging Research Laboratory. Their radiology report included number of definite microhemorrhages and subcortical infarcts at the baseline scans, corresponding to small ( ≤ 10mm) areas of signal void with associated blooming artifact on T2^*^ sequence and round or ovoid subcortical, fluid-filled lesions with signs of restricted diffusion, respectively (Duering et al., 2023). The occurrence of ARIA-H with microhemorrhages, ARIA-H with superficial siderosis, and ARIA-E at the follow-up MRI scans were also documented in the radiology report (see their radiological definitions in the Introduction; Sperling et al., 2011). Readers were blinded to baseline SVD markers. Cases with disagreements between readers (*N* = 158) were resolved by consensus.

WMH were segmented with the HyperMapp3r algorithm (https://hypermapp3r.readthedocs.io/), a Bayesian 3D convolutional neural network with a U-Net architecture, as previously described (Sperling et al., 2023). WMH volume was log-transformed to reduce skewness.

PVS were segmented on T1-weighted images using a robust, fully-automated algorithm previously validated (Barisano et al., 2025). While the derived measurements were previously demonstrated to be highly robust and to have excellent inter-scanner reproducibility (intraclass-correlation coefficients ≥0.8 for all metrics), we cannot exclude that small lesions with vascular shape may be erroneously included in our PVS segmentation masks and that some PVS, especially at the boundary of the basal ganglia masks, may be missed by the segmentation algorithm (Barisano et al., 2025). All PVS segmentation masks were visually inspected for accuracy in a blind fashion and are available at https://gbarisano.shinyapps.io/pvs-dementia/ (project: “A4”).

WMH, WM-PVS, and BG-PVS volumes were normalized and converted to volume fractions of the total intracranial volume (Supplementary Table 2), as recommended (Barisano et al., 2021, 2022).

### PVS segmentation accuracy

We used the Human Connectome Project Young Adults (Van Essen et al., 2013) and Aging (Bookheimer et al., 2019) datasets to assess accuracy of our algorithm in segmenting PVS, as previous independent studies from these datasets have provided both PVS visual scores (*N* = 100, 53 females; age 28.3 ± 3.9; WMH volume: 31 ± 92 mm^3^; Sepehrband et al., 2019) and PVS manually refined segmentations (*N* = 158, 91 females; age 57.8 ± 15.5; WMH volume: 401 ± 1120 mm^3^; Chai et al., 2025). We applied our PVS segmentation algorithm (Barisano et al., 2025) to T1-weighted images downsampled from the native 0.8 isotropic resolution to 1 mm isotropic resolution, to ensure processing consistency with the A4 MRI dataset analyzed in this study. Agreement between the PVS volume fraction computed with our algorithm, the PVS visual scores assessed by two neuroradiologists (inter-rater reliability: 0.96; Sepehrband et al., 2019), and the PVS volume fraction calculated on PVS masks refined by two neuroradiologists and one medical student trained by them (inter-rater reliability and DICE similarity index above 0.94; Chai et al., 2025) were assessed with Spearman's correlations. We then assessed voxel-wise overlap between PVS masks obtained with our algorithm and those manually refined. Masks were rigidly co-registered with nearest-neighbor interpolation. We incorporated a three-voxel tolerance margin into the overlap analysis to mitigate the effects of potential mask misalignment during the registration and the partial volume artifacts inherent in manual segmentation. We then calculated for each subject the percentage of PVS voxels obtained with our algorithm overlapping with the expert-defined PVS voxels (precision) as well as the percentage of expert-defined PVS voxels correctly identified as PVS by the algorithm (sensitivity). Finally, we measured the Spearman correlation between the volume of the individual PVS segmented with both approaches to further estimate the agreement in size at the individual PVS level.

### Statistical analysis

All the models described below were adjusted for the following baseline factors, as reported on the documented radiology report, clinical assessment, and subject health history: age, gender, race, body mass index, APOE genotype, daily smoking and alcohol consumption statuses, treatment group, history of cardiovascular disease, total cholesterol level, Hemoglobin A1C level, intracranial volume and amyloid load. Potential confounders were selected based on the modified disjunctive cause criterion, identifying demographic and clinical variables that have an association with the exposure and/or the outcome, as supported by findings from the scientific literature.

The association of SVD markers at the baseline with subsequent risk of developing ARIA-H with microhemorrhages, ARIA-H with superficial siderosis, or ARIA-E was assessed with Cox proportional-hazards models. Person-time was calculated in each participant from the baseline MRI scan (origin and start times) until the MRI where the new ARIA were documented or the last MRI, whichever occurred first (end time). The proportional-hazards assumptions were verified by assessing the relationship between Schoenfeld residuals and time.

Additionally, penalized splines with third-degree polynomials (cubic splines) as basis functions and two boundary knots corresponding to the minimal and maximal value of the marker and 8 equidistant interior knots were employed to flexibly model potential non-linear associations while minimizing the risk of overfitting. The rationale for this approach is that relationships between imaging biomarkers and clinical outcomes are often not strictly linear; imposing a linear functional form may obscure important threshold effects, plateaus, or inflection points. Cubic splines allow the data to inform the shape of the association by fitting smooth, piecewise polynomial functions joined at knots, while the penalization term constrains excessive curvature and reduces model complexity. This approach therefore provides a data-driven assessment of whether the exposure–outcome relationship deviates meaningfully from linearity. If the estimated spline function approximates a straight line, the association can be interpreted as largely linear across the observed range. Conversely, significant curvature may indicate the presence of thresholds, saturation effects, or differential risk gradients at low vs. high levels of the predictor. The effective degrees of freedom and visual inspection of the smooth term further inform the degree of non-linearity. Overall, the use of penalized cubic splines enhances interpretability by balancing flexibility and parsimony, allowing for a more accurate characterization of the underlying biological relationship without imposing restrictive assumptions.

The chi-square testing for zero slope in a regression of the spline coefficients on the centers of the basis functions was then used to assess the linearity of the association. For each marker showing statistically significant associations with increased risk of ARIA, we identified the value at which the spline-estimated hazard ratio crossed the reference value 1. This value was defined as the threshold. For continuous markers, the threshold was estimated with decimal precision using model-based predictions. For discrete markers restricted to integer values (e.g., number of microhemorrhages), the threshold was operationalized as the first observed integer value with spline-estimated hazard ratio above the reference value 1. To evaluate the robustness of the identified thresholds, 500 simulations were generated with non-parametric bootstrap iteration to estimate threshold values indicative of increased risk. In each iteration, the spline model was refitted and the threshold re-estimated. The distribution of bootstrap-derived thresholds was examined to assess consistency and stability. Sensitivity analyses were performed to assess the influence of other potential confounding factors to the main results, including: (1) tau status (available in 509 participants) and (2) MRI scanner manufacturer. We also examined whether the results of the primary Cox proportional-hazards model for WM-PVS were robust to the exclusion of potential false-positive WM-PVS adjacent to white matter hyperintensities. These voxels were excluded from the WM-PVS binary masks by subtracting the WMH mask after three-dimensional isotropic dilation by 2 voxels. The WM-PVS volume of the remaining voxels was measured and used in the Cox proportional-hazards model. Finally, we assessed whether the accuracy of PVS segmentation could influence the results of the primary Cox proportional-hazards model by including an accuracy rating score as a covariate in the statistical model. The accuracy rating score was generated by a single rater with more than 10 years of experience in neuroimaging and PVS analysis who classified the segmentations as “no/minimal inaccuracy” (less than 10 PVS mis-segmented, including both false positive and false negative PVS), “mild inaccuracy” (between 10 and 20 PVS mis-segmented) and “moderate inaccuracy” (more than 20 PVS mis-segmented). The intra-rater correlation coefficient assessed on 30 random cases rated twice in a blinded fashion 1 month apart was very good (ICC = 0.81).

Subgroup analyses were performed for each SVD marker to explore effect modifications across levels of age, gender, overweight/obese status (body mass index ≥ 25), APOE4 status, and the baseline level of the other relevant SVD markers assessed.

To account for multiple testing, P values were adjusted with the Benjamini-Hochberg procedure (Benjamini and Hochberg, 1995). All statistical analyses were performed in R v4.4.3.

## Results

### PVS segmentation accuracy

**Figure 2:**
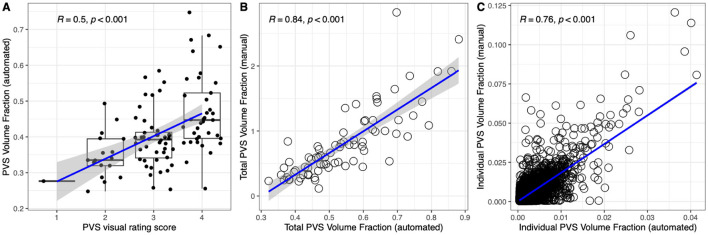
PVS segmentation accuracy. There was a significant positive correlation **(A)** between PVS voxels identified with our fully automated technique (y-axis) and the PVS visual scores assigned by expert neuroradiologists (x-axis; *N* = 100). Consistently, PVS volume measured with our method also showed a significant positive correlation with the PVS volume computed from masks refined by experts [**(B)**; *N* = 158]. Across individual PVS consistently segmented with both approaches (*N* = 12,713), there was a strong positive correlation between the PVS volumes computed with the two approaches **(C)**. R is the Spearman's ρ.

We previously showed that the PVS measurements obtained with our algorithm were strongly associated with age, sex, body mass index, cardiovascular risk factors, and cognitive status (Barisano et al., 2025), replicating results well-established within the PVS scientific literature (Barisano et al., 2022; Wardlaw et al., 2020). Here, we performed additional analyses to further assess the accuracy of our segmentation approach. We visually assessed the PVS segmentations: 76% of cases presented no/minimal PVS segmentation inaccuracy, 22% mild segmentation inaccuracy, and 2% moderate segmentation inaccuracy. We found that the PVS volume computed by our algorithm was significantly correlated with PVS visual scores as well as with the PVS volume of expert-defined PVS masks (Figures 2A, B). The automated algorithm achieved a good precision level, with a mean spatial overlap of 69 ± 16% with the expert-defined PVS masks. Concerning sensitivity, 89 ± 14% of the human-rated PVS voxels were correctly identified by the algorithm. When considering the size of the individual PVS consistently segmented with both approaches (*N* = 12,713 PVS), there was a strong correlation between the PVS volumes of each individual PVS segmented with the two approaches (Figure 2C). Overall, these data show that our fully-automated algorithm is accurate, providing results consistent with those achieved by experts.

### Association between imaging markers and ARIA risk

Among 1,165 participants included in the trial, 77 cases were excluded due to the lack of a brain MRI scan available at the baseline (46 cases) or due to missing information on clinical covariates of interest (31 cases), resulting in a final sample size of 1,088 participants (Table 1 and Supplementary Figure 1). No relevant differences in clinical and demographic characteristics were observed between the excluded cases and those included (Supplementary Table 3).

**Table 1:** Baseline characteristics of the study population.

Characteristic	Overall *N* = 1,088^a^	No ARIA *N* = 732^a^	ARIA *N* = 356^a^	*P-*value^b^
Baseline clinical and demographic data				
Age (years)	71.0 (68.0,75.0)	70.8 (67.9,75.0)	71.4 (68.1,75.1)	0.3
Gender				0.001
Male	442 (40.6%)	273 (37.3%)	169 (47.5%)	
Female	646 (59.4%)	459 (62.7%)	187 (52.5%)	
Race				0.6
American Indian or Alaskan Native	1 (0.1%)	0 (0.0%)	1 (0.3%)	
Asian	23 (2.1%)	13 (1.8%)	10 (2.8%)	
Black or African American	26 (2.4%)	18 (2.5%)	8 (2.2%)	
More than one race	8 (0.7%)	6 (0.8%)	2 (0.6%)	
Not Reported	6 (0.6%)	5 (0.7%)	1 (0.3%)	
White	1,024 (94.1%)	690 (94.3%)	334 (93.8%)	
Education (years)	16.0 (15.0, 18.0)	16.0 (15.0, 18.0)	16.0 (16.0, 18.0)	>0.9
Body Mass Index (kg/m^2^)	26.6 (24.0, 30.0)	26.6 (23.9, 30.2)	26.5 (24.1, 29.7)	0.7
APOE				<0.001
E3/E3	391 (35.9%)	287 (39.2%)	104 (29.2%)	
E2/E2	1 (0.1%)	1 (0.1%)	0 (0.0%)	
E2/E3	58 (5.3%)	43 (5.9%)	15 (4.2%)	
E2/E4	34 (3.1%)	26 (3.6%)	8 (2.2%)	
E3/E4	512 (47.1%)	334 (45.6%)	178 (50.0%)	
E4/E4	92 (8.5%)	41 (5.6%)	51 (14.3%)	
Daily alcohol consumption status	552 (50.7%)	363 (49.6%)	189 (53.1%)	0.3
Daily smoking status	20 (1.8%)	13 (1.8%)	7 (2.0%)	0.8
Presence of cardiovascular disease	107 (9.8%)	69 (9.4%)	38 (10.7%)	0.5
Total serum cholesterol (mmol/L)	5.1 ± 1.0	5.2 ± 1.0	5.1 ± 1.1	0.6
HbA1C (%)	5.5 (5.3,5.8)	5.5 (5.3,5.8)	5.5 (5.3,5.8)	0.7
Amyloid burden on ^18^F-florbetapir PET (Centiloid)	66.1 ± 32.8	64.6 ± 32.8	69.3 ± 32.7	0.016
Tau status				0.6
Negative	314 (61.7%)	207 (62.5%)	107 (60.1%)	
Positive	195 (38.3%)	124 (37.5%)	71 (39.9%)	
Not available	579	401	178	
ARIA-H with Microhemorrhages	342 (31.4%)	0 (0.0%)	342 (96.1%)	<0.001
ARIA-H with Superficial Siderosis	40 (3.7%)	0 (0.0%)	40 (11.2%)	<0.001
ARIA-E	4 (0.4%)	0 (0.0%)	4 (1.1%)	0.011
SVD features on baseline MRI scan				
Microhemorrhages				<0.001
0	886 (81.4%)	641 (87.6%)	245 (68.8%)	
1	146 (13.4%)	74 (10.1%)	72 (20.2%)	
2	39 (3.6%)	14 (1.9%)	25 (7.0%)	
3	8 (0.7%)	1 (0.1%)	7 (2.0%)	
≥ 4	9 (0.8%)	2 (0.3%)	7 (2.0%)	
Subcortical infarcts				0.8
0	962 (88.4%)	651 (88.9%)	311 (87.4%)	
1	99 (9.1%)	64 (8.7%)	35 (9.8%)	
2	23 (2.1%)	15 (2.0%)	8 (2.2%)	
3	2 (0.2%)	1 (0.1%)	1 (0.3%)	
≥ 4	2 (0.2%)	1 (0.1%)	1 (0.3%)	
WMH volume (cm^3^)	3.3 (1.7, 6.4)	2.9 (1.5, 5.8)	4.0 (2.0, 7.6)	<0.001
WM-PVS volume (cm^3^)	2.3 (1.9, 2.8)	2.3 (1.9, 2.8)	2.4 (1.9, 2.8)	0.067
BG-PVS volume (mm^3^)	429.0 (394.0, 467.0)	428.0 (391.5, 467.0)	433.0 (400.0, 468.0)	0.2
WMH volume fraction (log)	3.1 (2.5, 3.8)	3.0 (2.4, 3.7)	3.3 (2.6, 3.9)	<0.001
WM-PVS volume fraction	1.6 (1.4, 1.9)	1.6 (1.3, 1.9)	1.6 (1.4, 2.0)	0.3
BG-PVS volume fraction	30.3 (28.2, 32.6)	30.3 (28.2, 32.7)	30.3 (28.1, 32.6)	0.5

^a^Median (Q1,Q3); *n* (%); Mean ± SD.

^b^Wilcoxon rank sum test for continuous variables; Pearson's Chi-squared test for categorical variables, except for “Race”, “APOE”, and “ARIA-E” where Fisher's exact test was used. Race and gender were reported by the participants. Cardiovascular disease includes any of the following: heart failure, angina, cardiac arrest, stent placement, coronary artery bypass, pacemaker, defibrillator, heart valve replacement or repair, stroke, transient ischemic attack. “Not available” in “Tau status” means that these subjects did not undergo a Tau PET scan nor a lumbar puncture for assessment of Tau biomarkers.

**Figure 3:**
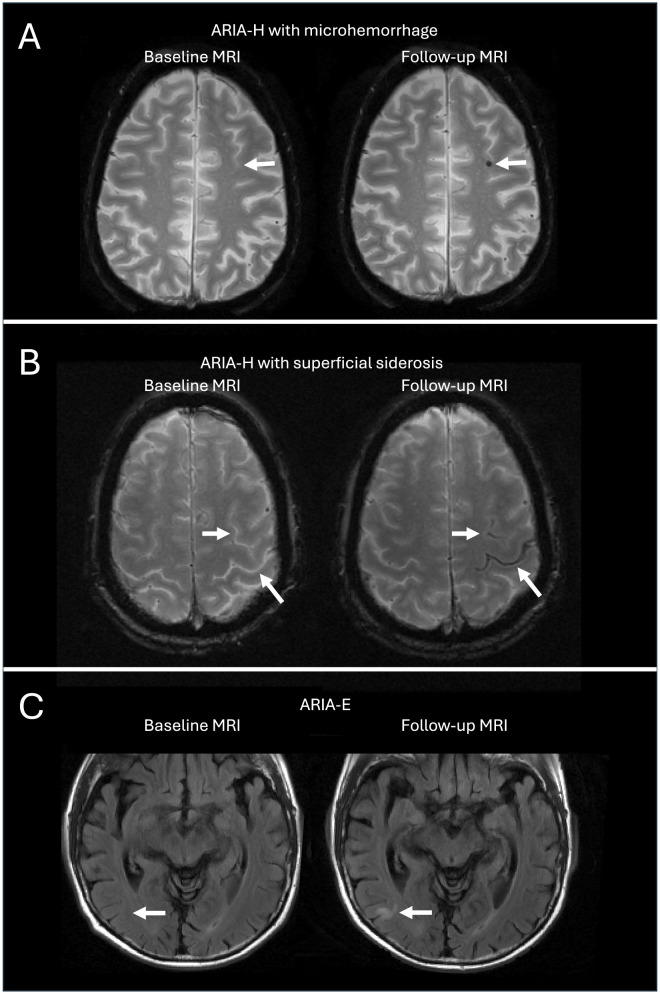
Representative images of ARIA in the A4 study. **(A)** ARIA-H with microhemorrhage appears as small parenchymal areas of signal void with associated blooming artifact on T2-star sequence. **(B)** ARIA-H with superficial siderosis is visible as hypointense signal in the leptomeninges on T2-star sequence. **(C)** ARIA-E refers to parenchymal edema manifesting as hyperintensities on T2-weighted FLAIR sequence. Axial T2-star **(A, B)** and FLAIR **(C)** images of the brain obtained before (left, “Baseline MRI”) and after (right, “Follow-up MRI”) the development of ARIA. In each panel, white arrows indicate the same cerebral area in the two MRI scans.

During a median study follow-up of 5.4 years (interquartile range, 4.1–5.7), 6,028 brain MRI scans were acquired (average of 5.5±1.6 scans per subject) at an average interval of 11.8±3.1 months between scans: a total of 356 ARIA events (incidence rate, 59.6 events per 1000 person-years) were diagnosed, including 342 ARIA-H with microhemorrhages, 40 ARIA-H with superficial siderosis, and 4 ARIA-E (Figure 3).

**Figure 4:**
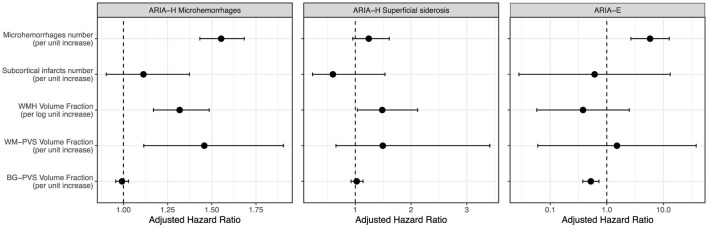
Forest Plots for the Associations of small vessel disease imaging markers with ARIA Risk. Each additional microhemorrhage, logarithmic unit of WMH volume fraction, and unit of WM-PVS volume fraction was associated with 55, 32, and 46% increase in ARIA-H with microhemorrhages risk, respectively **(left panel)**. Each additional logarithmic unit of WMH volume fraction was also associated with 48% increase in ARIA-H with superficial siderosis risk **(central panel)**. Each additional microhemorrhage and unit of BG-PVS volume fraction were associated with 5.8 times increase and 48% decrease in ARIA-E risk, respectively **(right panel)**. Subcortical infarcts were not associated with ARIA risk. Hazard ratios have been adjusted for potential confounding factors, including age, gender, race, body mass index, APOE genotype, daily smoking and alcohol consumption statuses, treatment group, history of cardiovascular disease, total cholesterol level, Hemoglobin A1C level, intracranial volume and amyloid load.

Each additional microhemorrhage, logarithmic unit of WMH volume fraction, and unit of WM-PVS volume fraction was associated with 55% (adjusted hazard ratio, 1.55; 95% CI, 1.43–1.68), 32% (adjusted hazard ratio, 1.32; 95% CI, 1.17–1.48), and 46% (adjusted hazard ratio, 1.46; 95% CI, 1.12–1.91) increase in risk of ARIA-H with microhemorrhages, respectively (Figure 4). Each additional logarithmic unit of WMH volume fraction was also associated with 48% increase (adjusted hazard ratio, 1.48; 95% CI, 1.04–2.12) in ARIA-H with superficial siderosis risk, whereas each additional microhemorrhage and unit of BG-PVS volume fraction were associated with 5.8 times increase (adjusted hazard ratio, 5.81; 95% CI, 2.67–12.65) and 48% decrease (adjusted hazard ratio, 0.52; 95% CI, 0.38–0.72) in ARIA-E risk, respectively (Figure 4). The number of subcortical infarctions was not associated with ARIA risk. All raw results from this analysis are included in the Supplementary File 2.

**Figure 5:**
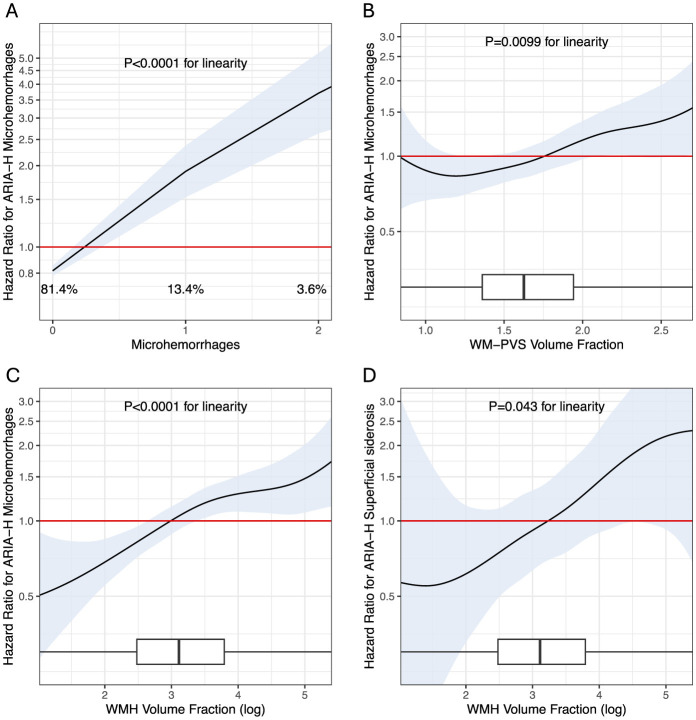
Spline plots for the associations of baseline microhemorrhages, WM-PVS volume fraction, and WMH volume fraction with ARIA-H Risk. The spline analysis supported a linear association over the range of microhemorrhages count **(A)**, WM-PVS volume fraction **(B)**, and WMH volume fraction **(C, D)**. Shaded areas indicate 95% confidence intervals, and the red line at 1.0 indicates the reference. At the bottom of the graphs, percentages (Panel A) and box plots **(B–D)** show the distributions of the marker in the study population. In the box plots, the vertical bar indicates the median, and the ends of the box the interquartile range; the whiskers extend to values no farther than 1.5 times the interquartile range (which may be past the graphed area). *P* indicate *P*-values from the chi-square test for linearity adjusted for multiple testing. See also Supplementary Figures 2, 3 for sensitivity analysis. Hazard ratios have been adjusted for potential confounding factors, including age, gender, race, body mass index, APOE genotype, daily smoking and alcohol consumption statuses, treatment group, history of cardiovascular disease, total cholesterol level, Hemoglobin A1C level, intracranial volume and amyloid load.

The spline analysis supported a significant linear association over the range of the microhemorrhages, WMH, and WM-PVS measured at the baseline MRI (Figures 5A–C) and identified critical values of these markers indicating increased risk or protection for ARIA-H: having ≥1 microhemorrhages, ≥1.75 WM-PVS volume fraction, or ≥3 log-units of WMH volume fraction is associated with higher risk of ARIA-H with microhemorrhages, whereas values below those thresholds are associated with lower risk (Figures 5A–C); having ≥3.2 log-units of WMH volume fraction is also associated with higher risk of ARIA-H with superficial siderosis (Figure 5D). These values were further confirmed via bootstrapping with 500 iterations (Supplementary Table 4).

Sensitivity analyses showed substantially unchanged results, suggesting that these effects are independent of other potential confounding factors, including the MRI scanner (Supplementary Figure 2) and the positivity status for tau (Supplementary Figure 3), were not influenced by the potential false positive WM-PVS adjacent/within WMH (Supplementary Figure 4A), and were not driven by the cases with some inaccuracies identified in the PVS segmentations (Supplementary Figure 4B).

**Figure 6:**
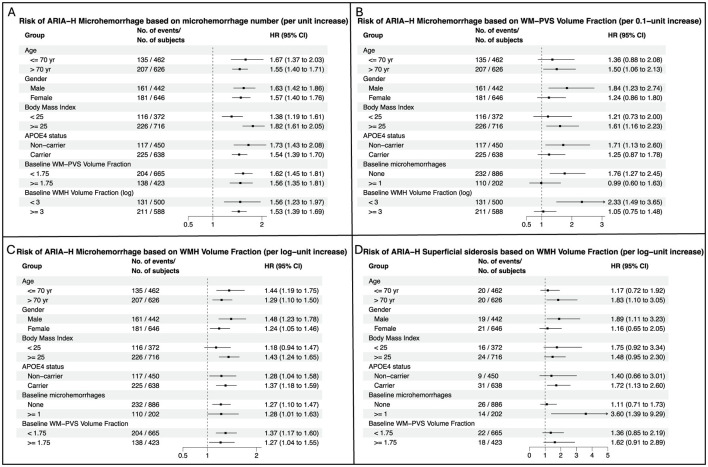
Subgroup analyses for the associations of baseline microhemorrhages, WM-PVS volume fraction, and WMH volume fraction with ARIA-H Risk. **(A)** Associations of baseline microhemorrhages with subsequent risk of ARIA-H with microhemorrhages. **(B)** Associations of baseline WM-PVS volume fraction with subsequent risk of ARIA-H with microhemorrhages. **(C)** Associations of baseline WMH volume fraction with subsequent risk of ARIA-H with microhemorrhages. **(D)** Associations of baseline WMH volume fraction with subsequent risk of ARIA-H with superficial siderosis. In each panel, hazard ratios have been adjusted for potential confounding factors, including age, gender, race, body mass index, APOE genotype, daily smoking and alcohol consumption statuses, treatment group, history of cardiovascular disease, total cholesterol level, Hemoglobin A1C level, intracranial volume, and amyloid load.

Subgroup analyses revealed that the effects of baseline microhemorrhages and WHM volume fraction on the risk of ARIA-H with microhemorrhages were consistent across all subgroups, whereas the effect of baseline WM-PVS volume fraction on the risk of ARIA-H with microhemorrhages was observed in APOE4 non-carriers, in participants without baseline microhemorrhages or low WMH burden, in older subjects, in males, and in overweight/obese participants (Figures 6A–C). The effect of WMH volume on the risk of ARIA-H with superficial siderosis was found in older individuals, in males, in APOE4 carriers, and in individuals with ≥1 microhemorrhage at the baseline scan (Figure 6D). The corresponding Kaplan-Meier plots confirmed overall these findings (Supplementary Figure 5).

Given the low incidence of ARIA-E in this study, the spline model did not converge and it was not possible to perform the subgroup analysis for ARIA-E. Hence, the results for ARIA-E should be treated as hypothesis-generating only, no firm inference should be drawn. Supplementary Figure 6 depicts adjusted hazard ratios plotted over the range of the measured markers that were significantly associated with ARIA-E risk (i.e., baseline microhemorrhages and BG-PVS volume fraction).

## Discussion

In this study assessing 6,028 brain MRI scans acquired in 1,088 patients with preclinical Alzheimer's Disease enrolled in a phase-3 trial of solanezumab, the number of microhemorrhages and the volume fractions of WMH and WM-PVS assessed at the baseline MRI scan were associated with higher risk of subsequent ARIA-H with microhemorrhages in a dose–response manner. WMH were also associated with risk of ARIA-H with superficial siderosis, whereas baseline microhemorrhages number and BG-PVS volume fraction were associated with ARIA-E risk. Previous amyloid-modifying therapeutic trials evaluated the incidence and risk factors associated with ARIA. Specifically, some studies reported that the presence of microhemorrhages at baseline was associated with higher ARIA-H (Arrighi et al., 2016) and ARIA-E (Joseph-Mathurin et al., 2022; Salloway et al., 2022) risk, although limited number of confounding factors were considered and the dose-response relationship with their count was not assessed. In aducanumab, donanemab, and lecanemab phase-3 trials, participants with >4 microhemorrhages at the baseline were excluded from the trial (Salloway et al., 2022; Sims et al., 2023; van Dyck et al., 2023). Current clinical guidelines recommend this same threshold (Cummings et al., 2023). These results controlling for relevant confounding factors show that the risk of ARIA-H and ARIA-E is more than 50% higher even when a single microhemorrhage is detected on baseline MRI compared with no microhemorrhages, suggesting that decreasing the currently recommended threshold of 4 microhemorrhages may be a valid safer option to consider. Although previous studies did not report significant associations between incident ARIA and visual rating of WMH, possibly due to the lower sensitivity of the WMH visual assessment compared with quantitative evaluations of WMH (Arrighi et al., 2016; Sperling et al., 2012), participants with “diffuse/severe” white matter disease were excluded from recent trials (Salloway et al., 2022; Sims et al., 2023; van Dyck et al., 2023). Here a dose-response association between ARIA-H risk and WMH volume was identified. Additionally, the subgroup analysis indicated that the effect of baseline microhemorrhages and of WMH to ARIA-H risk are independent from each other, supporting the use of both markers when evaluating ARIA risk in patients. PVS were not previously investigated in the context of ARIA. WM-PVS were linearly associated with risk of ARIA-H with microhemorrhages. Interestingly, this effect was mostly observed in subgroups that are not considered at higher risk of ARIA, including APOE4 non-carriers and subjects with a low burden of WMH or without microhemorrhages, suggesting that WM-PVS enlargement may predispose these specific subgroups to ARIA-H via a pathophysiological mechanism potentially different than that occurring in APOE4 carriers and individuals with WMH or microhemorrhages. On the other hand, an inverse relationship was observed between BG-PVS volume fraction and ARIA-E risk. This finding is consistent with recent studies speculating that lower PVS amount may indicate PVS occlusion/collapse rather than enlargement, resulting in loss of fluid-like signal in PVS (hence decreased PVS detection on MRI) and altered cerebral fluid drainage and homeostasis (Barisano et al., 2025; Leone et al., 2025; Sepehrband et al., 2021). In fact, lower PVS amount in basal ganglia has been recently found associated with higher risk of cognitive decline and brain atrophy (Barisano et al., 2025). Lower PVS amount in the anterosuperior medial temporal lobe was also significantly associated with entorhinal neurofibrillary tau tangle deposition, a radiographic hallmark of early Alzheimer's disease pathology (Sepehrband et al., 2021). Moreover, lower BG-PVS count represents an early radiographic feature of autosomal dominant Alzheimer Disease independent of cardiovascular risk factors, preceding dementia diagnosis by almost two decades (Leone et al., 2025). The observed differential association of BG-PVS with ARIA-E and WM-PVS with ARIA-H also aligns with the distinct anatomical, physiological, and pathophysiological characteristics of PVS in these compartments: for example, BG-PVS are traditionally associated with vascular risk factors and hypertensive arteriopathy, whereas WM-PVS with amyloid burden, in particular CAA (Barisano et al., 2022; Giraud et al., 2025; Perosa et al., 2022; van Veluw et al., 2016, 2020; Wardlaw et al., 2020). These seemingly contrasting findings in BG-PVS and WM-PVS may actually be interconnected. Specifically, occlusion or reduction in BG-PVS could trigger a compensatory shift of perivascular fluid into the remaining open spaces of the WM-PVS. Further studies however are necessary to confirm these results and to validate these speculations.

PVS are normally visible in all individuals on current MRI scanners across the lifespan (Barisano et al., 2022), and represent a major element of the glymphatic system, a brain-wide system thought to be responsible for clearance of toxic and waste metabolites (Iliff et al., 2012). Alterations to PVS structure and morphology on MRI may be indicators of pathology affecting the cerebral blood vessels and/or the glymphatic system. Higher PVS volume fraction may indicate alterations in the glymphatic flow, with accumulation of fluid in PVS (Iliff et al., 2012). A recent study showed that larger PVS diameter is associated with higher dementia risk and accelerated brain atrophy in the non-demented elderly (Barisano et al., 2025). On the other hand, lower PVS visibility indicates lack of fluid-related signal on MRI, which in turn has been found associated with hypoperfusion (Barisano et al., 2025). This phenomenon may be related to PVS occlusion and/or alterations of PVS fluid flow due to impaired arterial pulsatility and vasomotion, major driving forces of fluid in PVS (Mestre et al., 2018; van Veluw et al., 2020). The positive association between WM-PVS volume fraction and ARIA-H with microhemorrhages identified in this study provides interesting insights about the potential pathophysiological mechanisms underlying ARIA. PVS alterations may be an indicator of different pathological entities contributing to ARIA, possibly inter-related to each other: (1) arteriolosclerosis, which in turn may result in endothelial damage ultimately leading to microhemorrhages and effusions; (2) perivascular inflammation, with increased vascular permeability, blood-brain barrier leakage, and/or microbleeds; (3) accumulation of amyloid and other proteins within and nearby the walls of blood vessels, weakening them and resulting in microhemorrhages; and (4) glymphatic dysfunction, with subsequently altered brain-fluid homeostasis and clearance of waste products.

PVS enlargement and WMH are recognized as complementary imaging markers of small vessel pathology in the context of CAA. In the Boston 2.0 criteria, both are included among the non-haemorrhagic neuroimaging markers that support the *in vivo* diagnosis of probable or possible CAA, reflecting the expanding view that CAA-related injury extends beyond strictly haemorrhagic manifestations (Charidimou et al., 2022). Their coexistence likely reflects overlapping but not identical pathophysiological processes, with important implications for disease staging, risk stratification, and treatment-related complications such as ARIA. PVS alterations, particularly in the centrum semiovale, are thought to reflect impaired interstitial fluid drainage along perivascular pathways (Barisano et al., 2022; Wardlaw et al., 2020). In CAA, amyloid-β deposition within cortical and leptomeningeal vessel walls may disrupt perivascular clearance. In fact, previous studies in patients with CAA have shown that WM-PVS dilation was associated with cortical CAA severity and vascular amyloid-β accumulation in the overlying cortex, but not with the presence of parenchymal amyloid plaques (Giraud et al., 2025; Perosa et al., 2022; van Veluw et al., 2016, 2020).

In contrast, WMH are generally interpreted as markers of chronic ischemic injury, demyelination, and gliosis related to small vessel dysfunction, although emerging evidence suggests that WMH may also represent a radiographic manifestation of non-vascular, AD-related white matter damage, as both tau and amyloid pathology may contribute to axonal degeneration (Garnier-Crussard, 2023). Thus, while WM-PVS alterations may be more specifically linked to CAA-related impaired perivascular clearance, WMH may represent downstream parenchymal injury from sustained vascular dysfunction and/or co-occurring AD-related processes. This is also supported by a recent study in patients with ADAD, where the prevalence of CAA ranges between 40% and 65% at autopsy (Ringman et al., 2016; Yin et al., 2025), showed that PVS structural alterations on MRI precede alterations in WMH, being detectable almost two decades before dementia diagnosis (Leone et al., 2025).

Our study has limitations. First, the number of incident ARIA-E cases diagnosed in this trial was relatively low, therefore the results related to ARIA-E need to be interpreted with caution. Second, given that clinical and MRI data from donanemab and lecanemab phase-3 trials are not made available to external investigators (Sims et al., 2023; van Dyck et al., 2023), this study was performed on data publicly available from a placebo-controlled trial of another anti-amyloid molecule, solanezumab, which showed lower efficacy in reducing amyloid-β burden (Sperling et al., 2023). Therefore, these results refer to spontaneous ARIA likely related to Alzheimer's Disease natural history rather than to the clearance of amyloid, although their underlying mechanisms may be similar considering that elements such as APOE4 genotype and microhemorrhages are consistently found as risk factors for ARIA in several amyloid-β targeting monoclonal antibodies, including lecanemab, donanemab, and solanezumab. Third, the potential role of residual unmeasured confounding factors that influence SVD burden and risk of cerebral hemorrhages, such as blood-pressure, antihypertensive/lipid-lowering medications, and diet, could not be ruled out. Fourth, participants included mainly white individuals without unstable medical conditions, so the generalizability of these results to other racial groups and to other patients with specific conditions is not possible. Fifth, given the close topological relationship between PVS and WMH, we cannot exclude that some voxels adjacent/within WMH and marked as PVS by our algorithm may in fact represent false positive PVS. However, the observed relationship between WM-PVS and risk of ARIA-H is supported by the consistent results obtained in a sensitivity analysis excluding voxels adjacent to WMH and by the results from the subgroup analysis showing that the relationship between WM-PVS and the risk of ARIA-H is evident in individuals with low WMH burden (<3 mL), suggesting that this relationship is related to true PVS voxels rather than to potential false positive PVS. Sixth, our approach for PVS segmentation did not allow to evaluate PVS inside WMH nor their topological relationship.

## Conclusions

This study showed a significant linear association in patients with preclinical Alzheimer's Disease between ARIA risk and quantitative measurements of SVD markers including number of microhemorrhages, WMH volume fraction, and PVS volume fraction, in a dose–response manner. These findings may help clinicians estimate an individual's risk of ARIA, potentially enabling patients and families to make informed treatment choices and enhancing the safety of clinical trials involving anti-amyloid therapies. Validation in the context of efficacious amyloid-targeting monoclonal antibody therapy such as lecanemab and donanemab is needed before the clinical implementation of these quantitative radiographic risk markers.

## Data Availability

The datasets presented in this study can be found in online repositories. The names of the repository/repositories and accession number(s) can be found below: https://www.a4studydata.org/; https://gbarisano.shinyapps.io/pvs-dementia/ (select project “A4”).
