# Supplementary material for: MRI evaluation of cerebral perivascular spaces predicts amyloid-related imaging abnormalities risk in preclinical Alzheimer's disease
Source: Front Dement. 2026 Apr 9;5:1719740. doi: 10.3389/frdem.2026.1719740 (PMC13102589; doi:10.3389/frdem.2026.1719740)
Supplement: Supplementary file 1 [file Supplementary_file_1.docx]

Supplemental Material

**Supplementary Table 1. Overview of the MRI scanners and parameters employed to acquire the images analyzed in the study.**

| Sequence | FS (T) | Manufacturer | TR  (sec) | TE  (sec) | TI (sec) | FA  (°) | Native resolution (mm^3^) | N (%) |
| --- | --- | --- | --- | --- | --- | --- | --- | --- |
| 3D T1-weighted imaging | 3 | GE | 0·007 | 0·003 | 0.4 | 11 | 1.2 x 1.1 x 1.1 | 254 (23.4) |
|  | 3 | Philips | 0·007 | 0·003 | 0.9 | 9 | 1.2 x 1.1 x 1.1 | 115 (10.6) |
|  | 3 | Siemens | 2.3 | 0.003 | 0.9 | 9 | 1.2 x 1.1 x 1.1 | 719 (66.1) |
| 2D Fluid Attenuated Inversion Recovery | 3 | GE | 11 | 0.154 | 2.25 | 90/125 | 0.9 x 0.9 x 5 | 254 (23.4) |
|  | 3 | Philips | 9 | 0.09 | 2.5 | 90 | 0.9 x 0.9 x 5 | 115 (10.6) |
|  | 3 | Siemens | 9 | 0.09 | 2.5 | 150 | 0.9 x 0.9 x 5 | 719 (66.1) |
| 2D T2* imaging | 3 | GE | 0.65 | 0.02 | - | 20 | 0.8 x 0.8 x 4 | 254 (23.4) |
|  | 3 | Philips | 0.65 | 0.02 | - | 20 | 0.8 x 0.8 x 4 | 115 (10.6) |
|  | 3 | Siemens | 0.65 | 0.02 | - | 20 | 0.8 x 0.8 x 4 | 719 (66.1) |

﻿FA: Flip Angle (degree, ◦); FS: Field Strength (Tesla, T); TE: Time to Echo (seconds); TI: Inversion Time (seconds); TR: Repetition Time (seconds).

**Supplementary Table 2. Scales of PVS and WMH volume fractions.**

| Marker | Normalization formula |
| --- | --- |
| WM-PVS | WM-PVS volume (mm^3^) / Intracranial volume (mm^3^) * 10^3^ |
| BG-PVS | BG-PVS volume (mm^3^) / Intracranial volume (mm^3^) * 10^5^ |
| WMH | logₑ [ WMH volume (mm^3^) / Intracranial volume (mm^3^) ] * 10^4^ |

﻿

**Supplementary Table 3. Comparison of the clinical and demographic characteristics of the patients excluded from the study and those included.**

| **Characteristic** | **Excluded** N = 81*^1^* | **Included** N = 1,088*^1^* | **p-value***^2^* |
| --- | --- | --- | --- |
| Age (years) | 72.2 (68.6, 75.0) | 71.0 (68.0, 75.0) | 0.2 |
| Gender |  |  | >0.9 |
| Male | 33 (40.7%) | 442 (40.6%) |  |
| Female | 48 (59.3%) | 646 (59.4%) |  |
| Race |  |  | 0.3 |
| American Indian or Alaskan Native | 1 (1.2%) | 1 (0.1%) |  |
| Asian | 1 (1.2%) | 23 (2.1%) |  |
| Black or African American | 2 (2.5%) | 26 (2.4%) |  |
| More than one race | 0 (0.0%) | 8 (0.7%) |  |
| Unknown or Not Reported | 1 (1.2%) | 6 (0.6%) |  |
| White | 76 (93.8%) | 1,024 (94.1%) |  |
| Education (years) | 16.0 (14.0, 18.0) | 16.0 (15.0, 18.0) | 0.13 |
| Body Mass Index (kg/m²) | 26.1 (23.7, 29.0) | 26.6 (24.0, 30.0) | 0.8 |
| Unknown | 2 | 0 |  |
| APOE |  |  | 0.045 |
| E3/E3 | 26 (32.1%) | 391 (35.9%) |  |
| E2/E2 | 1 (1.2%) | 1 (0.1%) |  |
| E2/E3 | 3 (3.7%) | 58 (5.3%) |  |
| E2/E4 | 1 (1.2%) | 34 (3.1%) |  |
| E3/E4 | 48 (59.3%) | 512 (47.1%) |  |
| E4/E4 | 2 (2.5%) | 92 (8.5%) |  |
| Daily Alcohol consumption status | 47 (58.8%) | 552 (50.7%) | 0.2 |
| Unknown | 1 | 0 |  |
| Daily Smoking status | 0 (0.0%) | 20 (1.8%) | 0.4 |
| Presence of Cardiovascular Disease | 6 (7.4%) | 107 (9.8%) | 0.5 |
| Total serum cholesterol (mmol/L) | 5.2 ± 0.9 | 5.1 ± 1.0 | 0.6 |
| Unknown | 4 | 0 |  |
| HbA1C (%) | 5.4 (5.3, 5.6) | 5.5 (5.3, 5.8) | 0.028 |
| Unknown | 4 | 0 |  |
| Amyloid burden on ¹⁸F-florbetapir PET (Centiloid) | 67.7 ± 32.9 | 66.1 ± 32.8 | 0.8 |
| Tau status |  |  | 0.6 |
| Negative | 19 (57.6%) | 314 (61.7%) |  |
| Positive | 14 (42.4%) | 195 (38.3%) |  |
| Unknown | 48 | 579 |  |
| ARIA Status |  |  | 0.14 |
| No ARIA | 61 (75.3%) | 732 (67.3%) |  |
| ARIA | 20 (24.7%) | 356 (32.7%) |  |
| ARIA-H with Microhemorrhages | 18 (22.2%) | 342 (31.4%) | 0.083 |
| ARIA-H with Superficial Siderosis | 5 (6.2%) | 40 (3.7%) | 0.2 |
| ARIA-E | 1 (1.2%) | 4 (0.4%) | 0.3 |

*^1^*Median (Q1, Q3); n (%); Mean ± SD.
*^2^* Wilcoxon rank sum test for continuous variables; Pearson's Chi-squared test for categorical variables, except for “Race”, “APOE”, and “ARIA-E” where Fisher's exact test was used.
Race and gender were reported by the participants. Cardiovascular disease includes any of the following: heart failure, angina, cardiac arrest, stent placement, coronary artery bypass, pacemaker, defibrillator, heart valve replacement or repair, stroke, transient ischemic attack.

|  |
| --- |

**Supplementary Table 4. Bootstrapping analysis with 500 iterations on risk thresholds for each marker.**

| Risk event | Baseline Marker | Threshold | Bias | Standard error |
| --- | --- | --- | --- | --- |
| ARIA-H with Microhemorrhage | Microhemorrhages number | 1 | -0.53 | 0.63 |
|  | WMH volume fraction (log) | 3.06 | -0.31 | 0.39 |
|  | WM-PVS  volume fraction | 1.83 | -0.36 | 0.17 |
| ARIA-H with  Superficial Siderosis | WMH volume fraction (log) | 3.2 | -0.48 | 0.37 |

Values indicate the estimated thresholds along with the bias and standard error estimated ﻿with 500 simulations generated with non-parametric bootstrap iterations for each marker.

**Supplementary Figure 1. Flow chart of the study participants.**


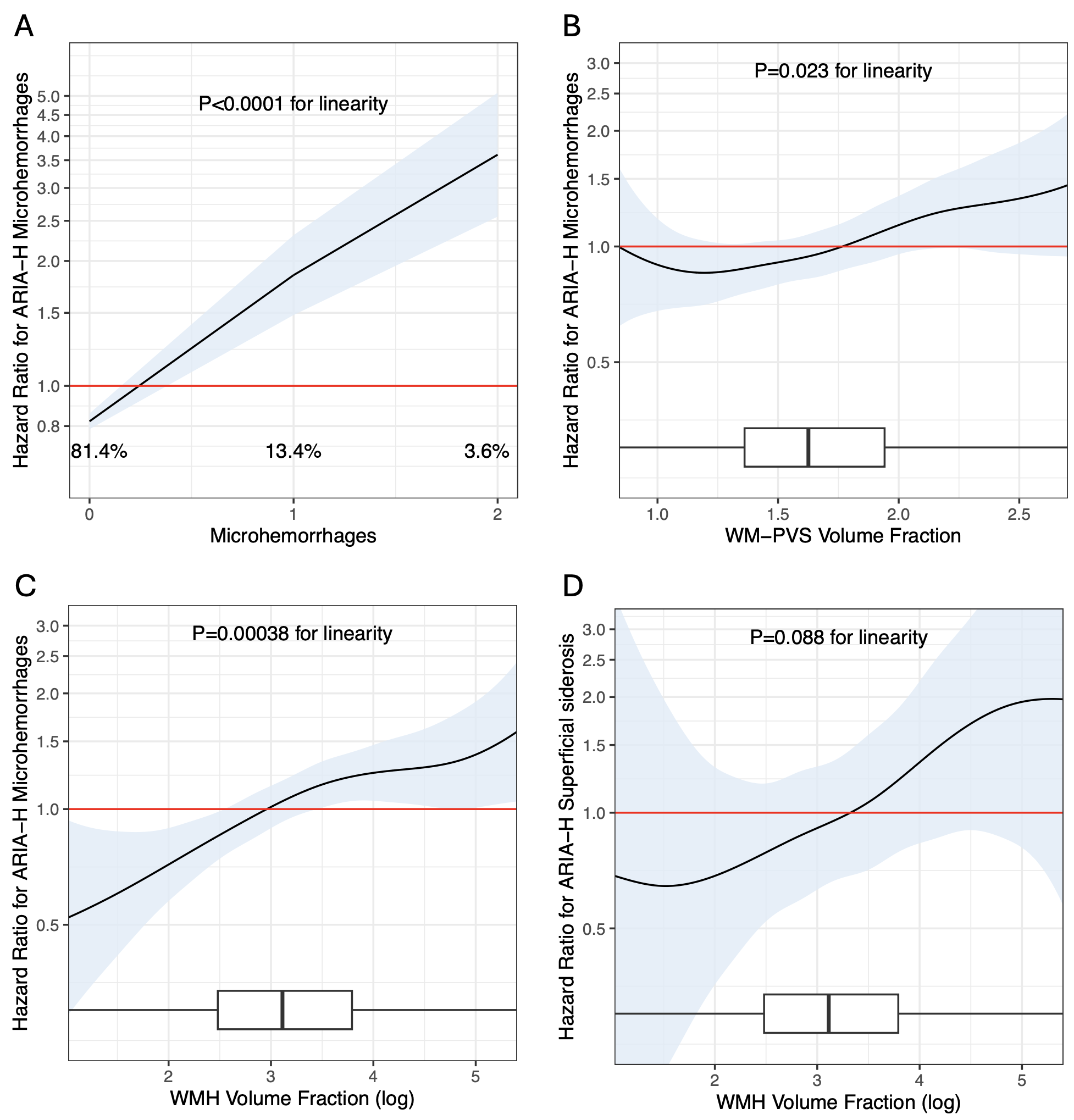


**Supplementary Figure 2. ﻿Spline Plots for the Associations of baseline microhemorrhages, WM-PVS volume fraction, and WMH volume fraction with ARIA-H Risk. Sensitivity analysis for MRI scanner manufacturer.**﻿The spline analysis supported a linear association over the range of microhemorrhages count (Panel A), WM-PVS volume fraction (Panel B), and WMH volume fraction (Panel C and D). Shaded areas indicate 95% confidence intervals, and the red line at 1.0 indicates the reference. At the bottom of the graphs, percentages (Panel A) and box plots (Panel B-D) show the distributions of the marker in the study population. In the box plots, the vertical bar indicates the median, and the ends of the box the interquartile range; the whiskers extend to values no farther than 1.5 times the interquartile range (which may be past the graphed area). P indicate P-values from the chi-square test for linearity adjusted for multiple testing. Hazard ratios have been adjusted for the same covariates of the main analysis in Figure 4, plus the MRI scanner manufacturer.


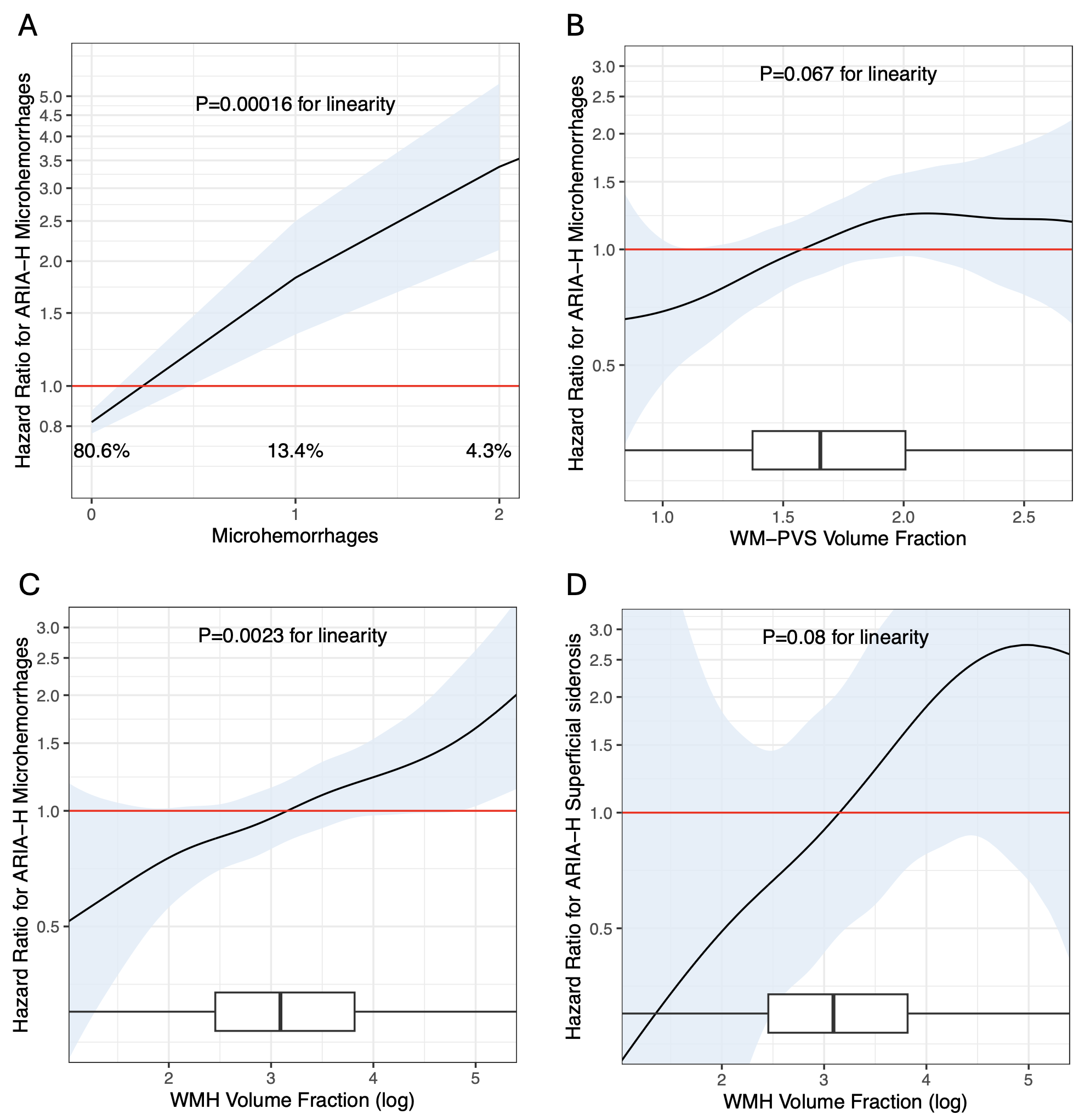


**Supplementary Figure 3. ﻿Spline Plots for the Associations of baseline microhemorrhages, WM-PVS volume fraction, and WMH volume fraction with ARIA-H Risk. Sensitivity analysis for tau status.**﻿The spline analysis supported a linear association over the range of microhemorrhages count (Panel A), WM-PVS volume fraction (Panel B), and WMH volume fraction (Panel C and D). Shaded areas indicate 95% confidence intervals, and the red line at 1.0 indicates the reference. At the bottom of the graphs, percentages (Panel A) and box plots (Panel B-D) show the distributions of the marker in the study population. In the box plots, the vertical bar indicates the median, and the ends of the box the interquartile range; the whiskers extend to values no farther than 1.5 times the interquartile range (which may be past the graphed area). P indicate P-values from the chi-square test for linearity adjusted for multiple testing. Hazard ratios have been adjusted for the same covariates of the main analysis in Figure 4, plus the tau-positivity status, which was available in 509 participants.

**Supplementary Figure 4. Forest Plots for the Associations of WM-PVS with ARIA Risk (sensitivity analysis).** In this sensitivity analysis, WM-PVS volume fraction was calculated after excluding potential false positive WM-PVS adjacent/within WMH (panel A) and after accounting for the PVS segmentation accuracy rating as a covariate in the statistical model (panel B). The results are consistent with the main model. Hazard ratios have been adjusted for potential confounding factors, including age, gender, race, body mass index, APOE genotype, daily smoking and alcohol consumption statuses, treatment group, history of cardiovascular disease, total cholesterol level, Hemoglobin A1C level, intracranial volume and amyloid load.

**Supplementary Figure 5. Kaplan-Meier survival curves for ARIA-H with microhemorrhages and ARIA-H with superficial siderosis according to risk groups for baseline microhemorrhages, WMH, and WM-PVS volume fractions.** ﻿﻿

In each panel, top plot reports the Kaplan-Meier survival curves for the occurrence of ARIA-H with microhemorrhages (panels A-C) or ARIA-H with superficial siderosis (panel D) according to risk groups based on the following baseline markers and thresholds: ≥1 (red) or <1 (cyan) baseline microhemorrhage (panel A), ≥3 (red) or <3 (cyan) log-units of baseline WMH volume fraction (panels B and D), and ≥1.75 (red) or <1.75 (cyan) of baseline WM-PVS volume fraction. Thresholds were derived from adjusted Cox proportional-hazards models with penalized splines (Figure 4). Vertical tick marks on lines indicate times at which the patient was censored. Shaded areas indicate 95% confidence intervals. The P values were derived from a log rank test for comparison between the two risk groups. Bottom plot in each panel reports the raw cumulative incidence measured as percentage of events occurring in each corresponding risk group.


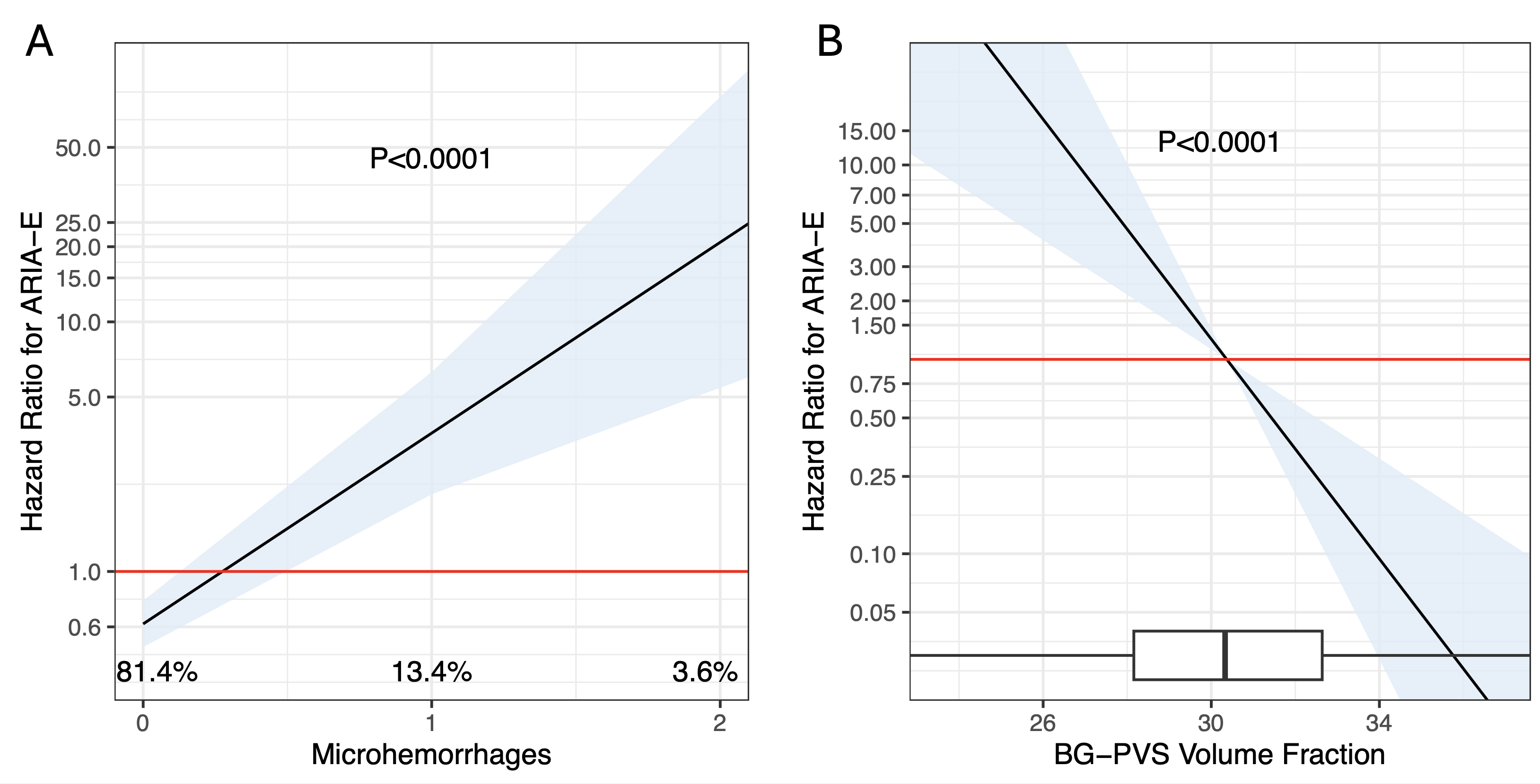


**Supplementary Figure 6. ﻿Associations of baseline microhemorrhages and BG-PVS volume fraction with ARIA-E Risk.**﻿These plots report the adjusted Hazard Ratios over the range of microhemorrhages count (Panel A) and BG-PVS volume fraction (Panel B) calculated with Cox proportional-hazards models adjusted for age, gender, race, body mass index, APOE genotype, daily smoking and alcohol consumption statuses, treatment group, history of cardiovascular disease, total cholesterol level, Hemoglobin A1C level, intracranial volume and amyloid load. Shaded areas indicate 95% confidence intervals, and the red line at 1.0 indicates the reference. At the bottom of the graphs, percentages (Panel A) and box plots (Panel B) show the distributions of the marker in the study population. In the box plots, the vertical bar indicates the median, and the ends of the box the interquartile range; the whiskers extend to values no farther than 1.5 times the interquartile range (which may be past the graphed area). P indicate P-values from the Cox proportional-hazards models adjusted for multiple testing.
